# Supplementary material for: The RNA-Binding Ubiquitin Ligase MEX3A Affects Glioblastoma Tumorigenesis by Inducing Ubiquitylation and Degradation of RIG-I
Source: Cancers (Basel). 2020 Jan 30;12(2):321. doi: 10.3390/cancers12020321 (PMC7072305; doi:10.3390/cancers12020321)
Supplement: Supplementary file 1 [file cancers-12-00321-s001.zip › cancers-690233-supplemenary-final/Supplementary Table S2.pdf]

Supplementary Table S2. Specific charcteristics of individual patient specimens.* In report.

Patients	Grade	Idh1	p53	EGFR
i#1	IV	Wild Type	Not expressed	Expressed
#2	IV	Mutated	Higly expressed	Not expressed
#3	IV	Wild Type	Expressed	Higly expressed
#4	IV	Wild Type	Expressed	n.d.
#5	III	Wild Type	Not expressed	n.d
#6	IV	Wild Type	Not expressed	Higly expressed
#7	IV	Wild Type	Not expressed	n.d.
#8	IV	Wild Type	Expressed	Not expressed
#9	IV	Wild Type	Not expressed	Higly expressed
#10	IV	Mutated	Expressed	Not expressed
#11	III	Wild Type	Expressed	n.d
#12	IV	Wild Type	Not expressed	Expressed
#13	IV	Wild Type	Very Low Expressed	Not expressed
#14	IV	Wild Type	Expressed	Not expressed
#15	IV	Wild Type	Expressed	Expressed
#16	IV	Wild Type	Very Low Expressed	Higly expressed
#17	IV	Wild Type	Expressed	Expressed
#18	IV	Wild Type	Not expressed	Higly expressed
#19	IV	Wild Type	Not expressed	Espressa
#20	IV	Wild Type	Expressed	Expressed
#21	IV	Wild Type	Expressed	Higly expressed
#22	IV	Wild Type	Not expressed	Expressed
#23	IV	Wild Type	Not expressed	Higly expressed
#24	IV	n.d.	Expressed	n.d.
#25 *	IV	n.d.	n.d.	n.d.
#26 *	IV	n.d.	n.d.	n.d.
#27 *	IV	n.d.	n.d.	n.d.

n.d. not determined.
