# Supplementary figures and images for: The RNA-Binding Ubiquitin Ligase MEX3A Affects Glioblastoma Tumorigenesis by Inducing Ubiquitylation and Degradation of RIG-I
Source: Cancers (Basel). 2020 Jan 30;12(2):321. doi: 10.3390/cancers12020321 (PMC7072305; doi:10.3390/cancers12020321)

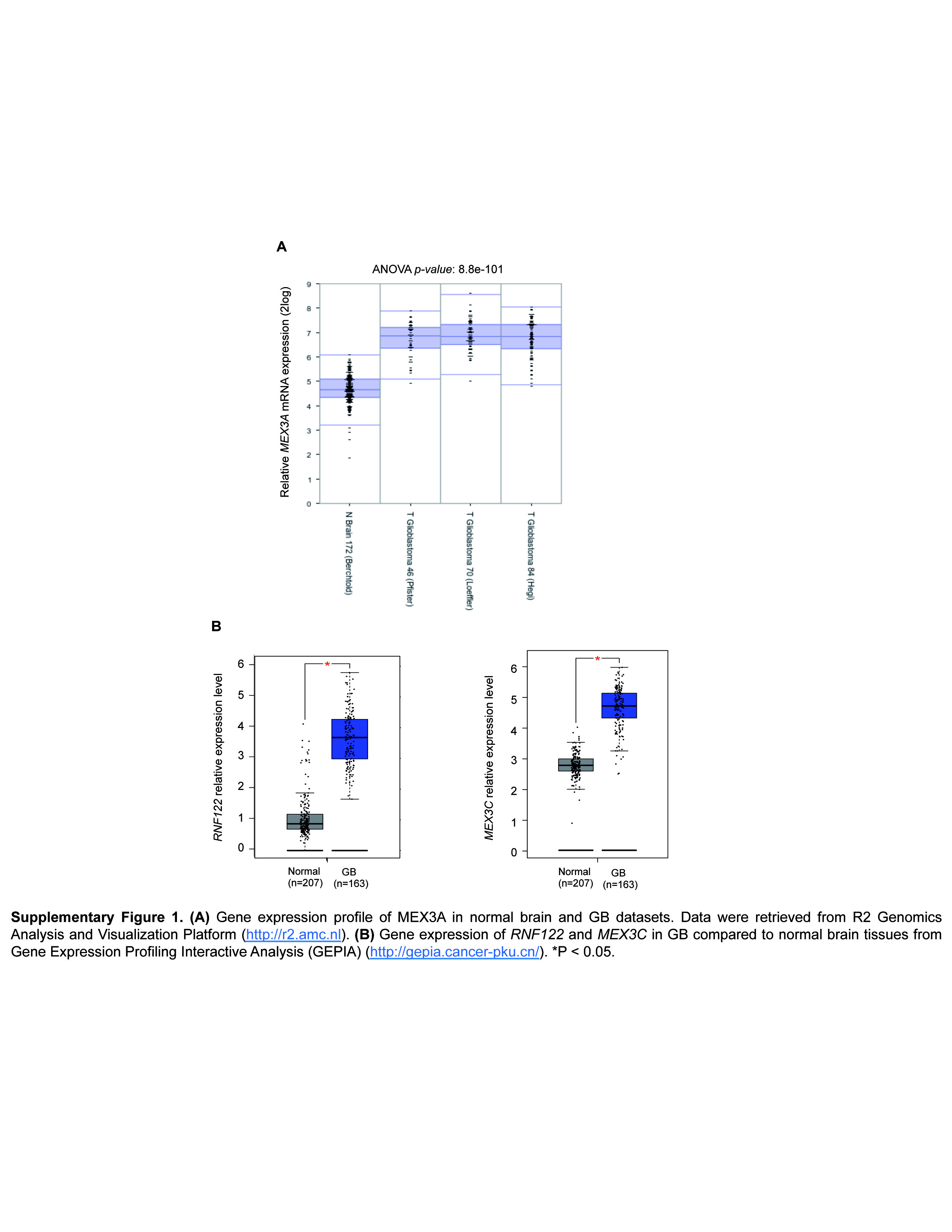

Supplement: Supplementary file 1 [file cancers-12-00321-s001.zip › cancers-690233-supplemenary-final/Figure S1.jpg]

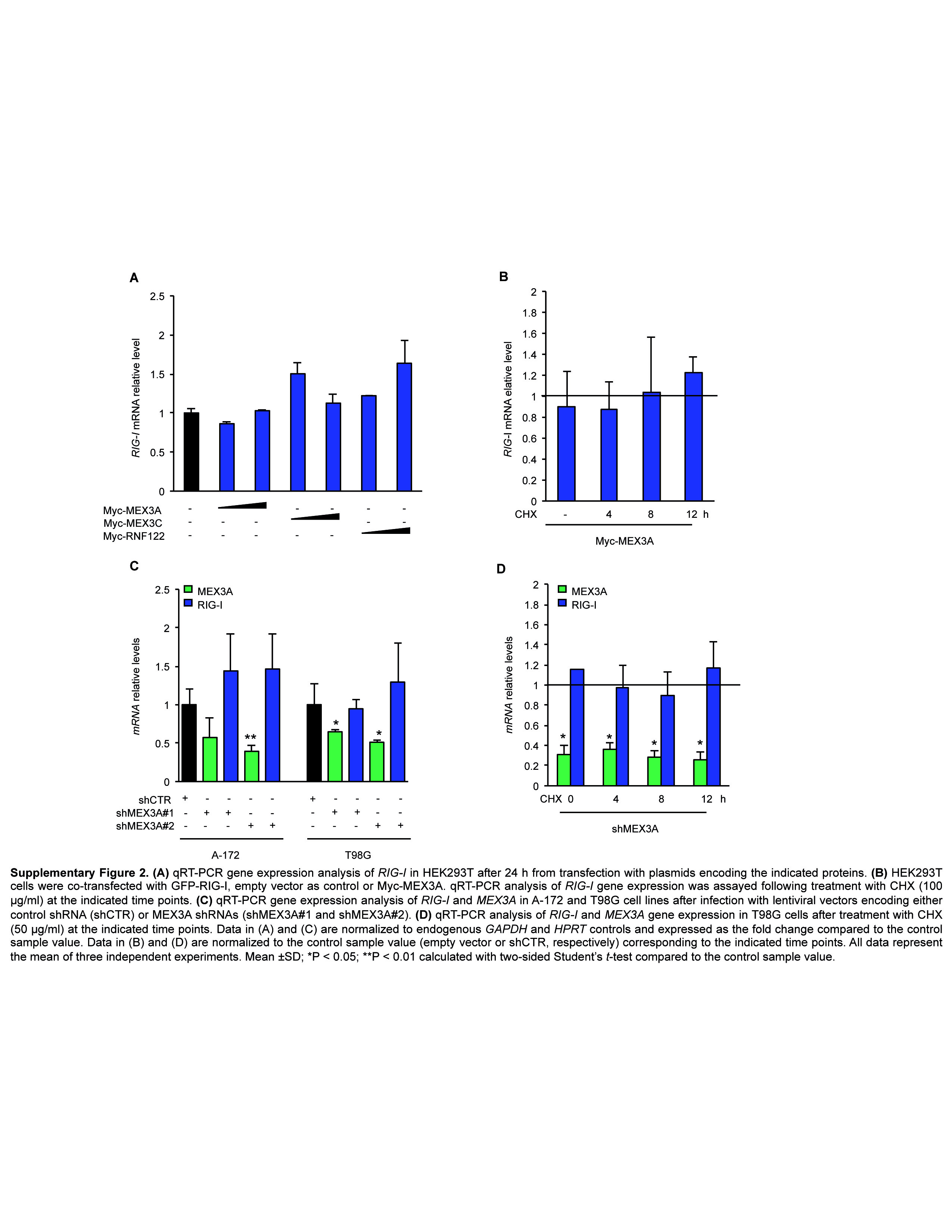

Supplement: Supplementary file 1 [file cancers-12-00321-s001.zip › cancers-690233-supplemenary-final/Figure S2.jpg]

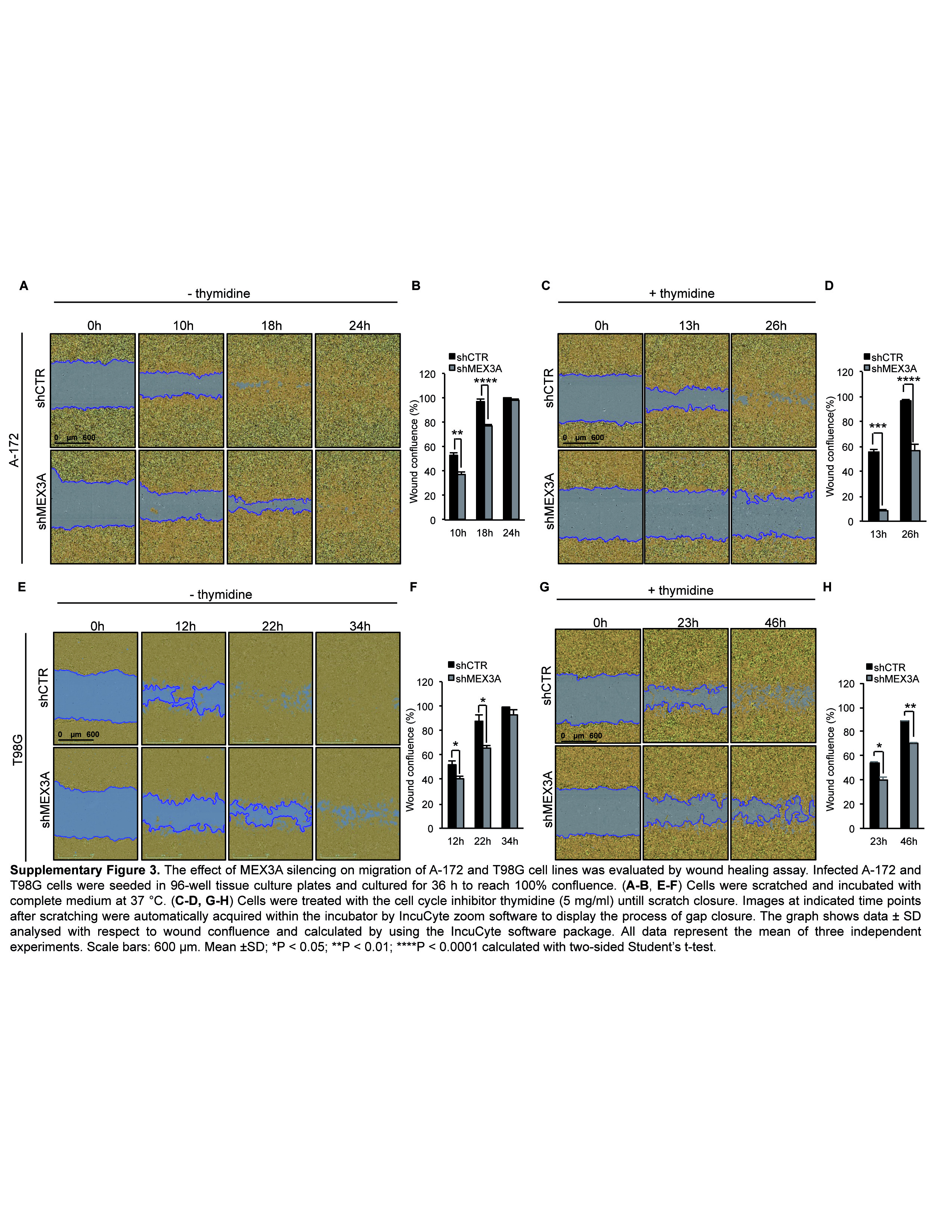

Supplement: Supplementary file 1 [file cancers-12-00321-s001.zip › cancers-690233-supplemenary-final/Figure S3.jpg]

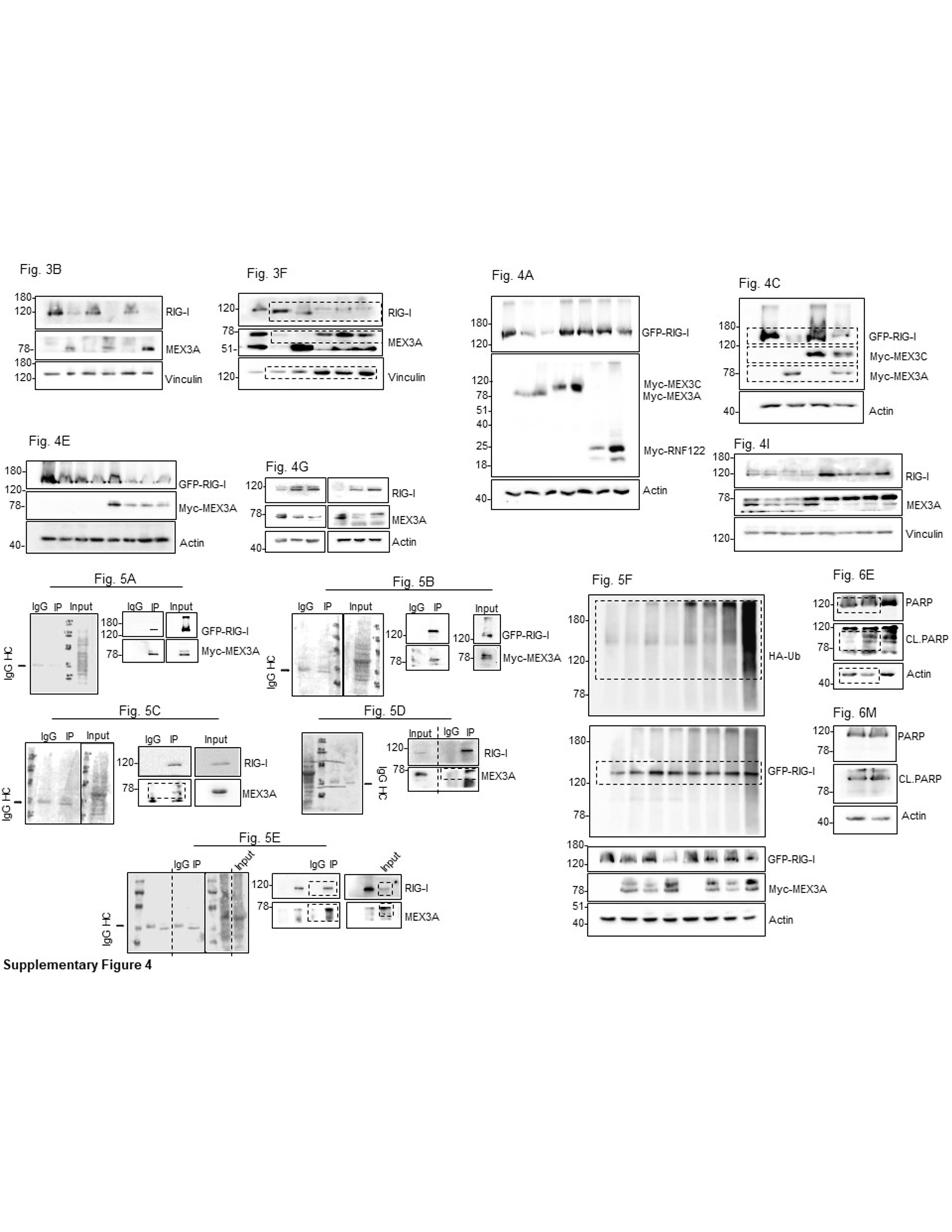

Supplement: Supplementary file 1 [file cancers-12-00321-s001.zip › cancers-690233-supplemenary-final/Figure S4.jpg]
